# SLTChemDB: A database of chemical compounds present in Smokeless tobacco products

**DOI:** 10.1038/s41598-019-43559-y

**Published:** 2019-05-09

**Authors:** Jasmine Kaur, Arun Sharma, Amit Kumar, Deeksha Bhartiya, Dhirendra Narain Sinha, Suchitra Kumari, Ruchika Gupta, Ravi Mehrotra, Harpreet Singh

**Affiliations:** 1 Informatics, Systems and Research Management, Indian Council of Medical Research (ICMR), New Delhi, 110029 India; 2 Data Management Laboratory, ICMR-National Institute of Cancer Prevention and Research, Noida, Uttar Pradesh 201301 India; 3 WHO FCTC Global Knowledge Hub of Smokeless Tobacco, National Institute of Cancer Prevention and Research, Noida, Uttar Pradesh 201301 India; 4 ICMR-National Institute of Cancer Prevention and Research, Noida, Uttar Pradesh 201301 India

**Keywords:** Chemical libraries, Cheminformatics, Databases, Cancer

## Abstract

Smokeless tobacco (SLT), a cause of potentially preventable diseases, has a diverse chemical composition encompassing toxicants as well as potent carcinogens. Though the chemical profile of SLT products has been analyzed earlier, this information is not available in a comprehensive and easily accessible format. Hence, there is an imperative felt need to develop a one-stop information source providing inclusive information on SLT products. SLTChemDB is the first such database that makes available detailed information on various properties of chemical compounds identified across different brands of SLT products. The primary information for the database was extracted through extensive literature search, which was further curated from popular chemical web servers and databases. At present, SLTChemDB contains comprehensive information on 233 unique chemical compounds and 82 SLT products. The database has been made user-friendly with facility for systematic search and filters. SLTChemDB would provide the initial data on chemical compounds in SLT products to various tobacco testing laboratories. The database also highlights research gaps and thus, would be a guide for researchers interested in chemistry and toxicology of SLT products. With regular update of information in the database, it shall be a valuable evidence base for policymakers to formulate stringent policies for SLT control.

## Introduction

Consumption of SLT is a global menace, estimated to account for approximately 0.65 million deaths per year^1^. Epidemiological studies indicate a significant role of SLT products in cancers, stroke, nervous and reproductive disorders^1–6^. A recent *in-silico* study indicated significant role of toxic chemical compounds in the diseases caused by SLT products^7^. Worldwide, there are different forms of SLT products available ranging from simple tobacco to complex products having many additives and flavoring agents. Reports suggest that the chemical composition of a tobacco plant gets altered significantly during the curing and processing of SLT products^8^. Many chemical compounds in the form of non-tobacco plant materials (like areca nut), humectants, flavoring agents and alkaline agents are also introduced to enhance the attractiveness and addictiveness of the SLT products^8^.

The chemical compounds identified so far in SLT products have been classified broadly into tobacco alkaloids, tobacco specific nitrosamines (TSNAs), volatile N-nitrosamines, N-nitrosamino acids, polycyclic aromatic hydrocarbons (PAHs), radionucleotides, metals, metalloids, pesticide residues, humectants, alfatoxins and mycotoxins^9–17^. TSNAs like N′-nitrosonornicotine (NNN), 4-(methylnitrosamino)-1-(3-pyridyl)-1-butanone (NNK), 4-(methylnitrosamino)-1-(3-pyridyl)-1-butanol (NNAL); PAHs like benzo[a]pyrene (B[a]P), radionuclides and alfatoxins have been classified as potent carcinogens by the International Agency for Research on Cancer (IARC)^6,18,19^. Nicotine, the predominant tobacco alkaloid and precursor of TSNAs, is also the cause of addictiveness associated with tobacco use^20–22^. Many independent studies have estimated the amount of nicotine, TSNAs, PAHs in various SLT products. Although existing studies estimated the chemical composition in a variety of products, there is a lack of adequate distribution and compilation of this pertinent information.

For effective control of SLT-attributable diseases, it is imperative to identify the chemical compounds present in SLT products, estimate their toxicity and study their specific role in diseases. No attempts have yet been made to compile data about the chemical compounds in SLT products. To the best of our knowledge, this study is the first attempt wherein we have collected and compiled vital details about the chemical information, physicochemical properties, biological information, toxicological information and distribution of chemical compounds present in SLT products. SLTChemDB is a one- stop information source crosslinked to various popular chemical databases like PubChem, ChemSpider and ChEMBL.

## Results

### Database Statistics

**Figure 1:**
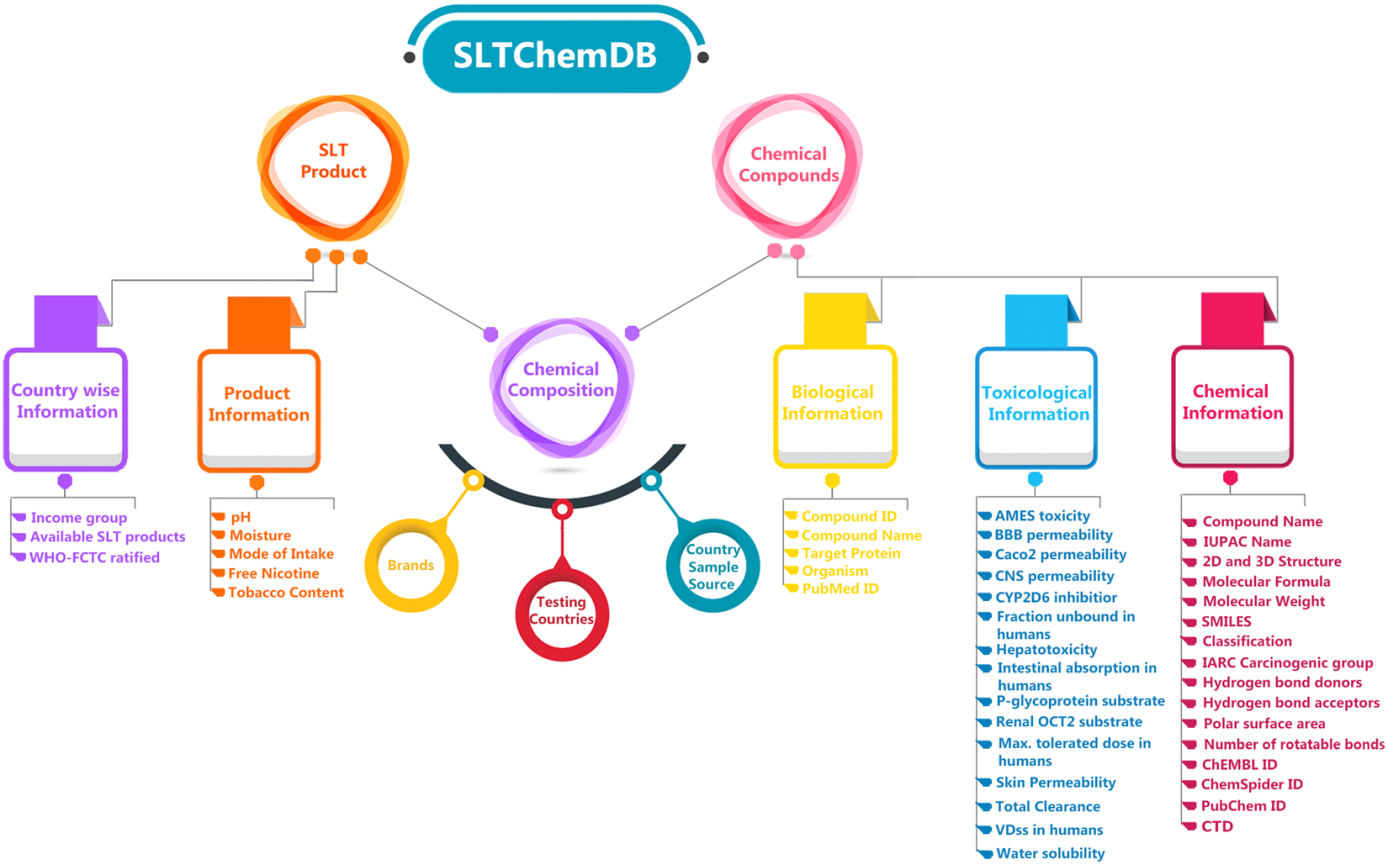
Schematic diagram depicting the information about SLT products and chemical compounds available in SLTChemDB.

SLTChemDB is a comprehensive database of all the chemicals identified by testing various SLT products. Presently, the database contains comprehensive information about 233 chemical compounds (+2 mixtures of these compounds) and 82 SLT products. We also provide brand-wise chemical composition, pH, moisture, free nicotine and tobacco content of 41 SLT products. Figure 1 briefly explains all the information available in SLTChemDB. The database contains information about chemical composition, pH, moisture, mode of intake, free nicotine, tobacco content and country-wise information of SLT products along with Biological, Toxicological and Physico-chemical information of chemical compounds.

Out of the 233 chemical compounds, chemical information like canonical SMILES and IUPAC name of 224 compounds were taken from PubChem^23^ and ChemSpider^24^. 3D structures/Canonical SMILES taken from PubChem/ChemSpider were converted to 2D using Open Babel^25^ (for more details refer to Supplementary Table 1). Structures of 5 compounds (unavailable on PubChem/ChemSpider) were self-drawn and SMILES were generated using the tool provided on the SLTChemDB website. Structures of 4 chemical compounds (2 PAH, 1 Coumarin and 1 Radionucleotide) could not be generated due to lack of complete chemical information. Canonical SMILES were used to calculate properties like polar surface area, number of donor and acceptor hydrogen bonds, molecular weight, molecular formula. Further, canonical SMILES was used to predict toxicological information using pkCSM web server^26^. Information about protein targets of 38 chemicals was extracted from ChEMBL^27^. Complete statistics of the chemical compounds is shown in Table 1.

**Table 1:** Table depicting overall statistics of chemical compounds present in SLTChemDB.

Total number of chemical compounds	233
Number of compounds having known physico-chemical properties	229
Number of compounds having known protein targets.	38
Number of compounds having known toxicological properties.	229
Number of carcinogenic compounds (as per IARC).	69
Number of SLT products	82

**Figure 2:**
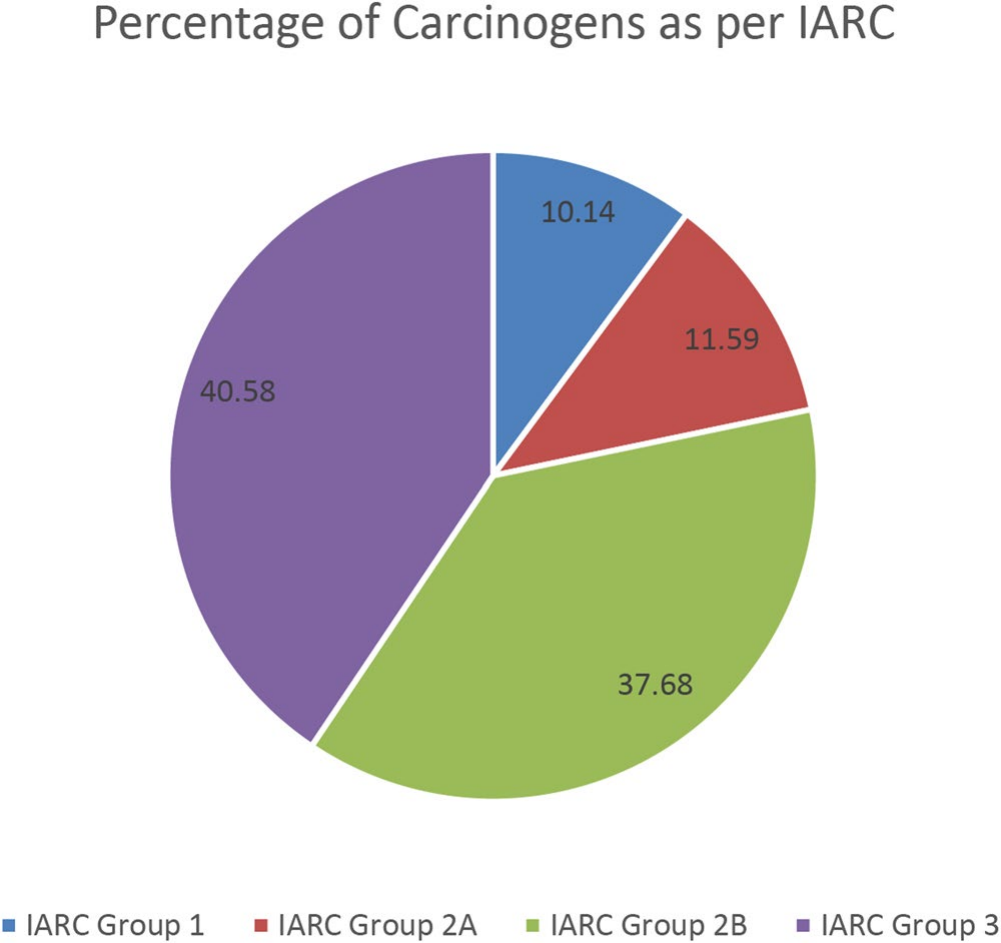
Pie chart depicting categorization of SLT compounds as per IARC classification of carcinogens.

As per the International Agency for Research on Cancer (IARC) classification of carcinogens^18^, 69 compounds out of the 233 compounds were classified various carcinogenic groups. Out of 69 classified compounds, 7 compounds (Formaldehyde, Beryllium, Arsenic, Cadmium, N-nitrosonornicotine, 4-(methylnitrosamino)-1-(3-pyridyl)-1- butanone, Benzo[a]pyrene) were classified as Carcinogenic to humans (IARC Group 1), 8 were classified as Probably Carcinogenic to humans (IARC Group 2 A), 26 were classified as Possibly Carcinogenic to humans (IARC Group 2B) and 28 were classified as not classifiable as per the carcinogenicity to humans (IARC Group 3) [Fig. 2].

**Figure 3:**
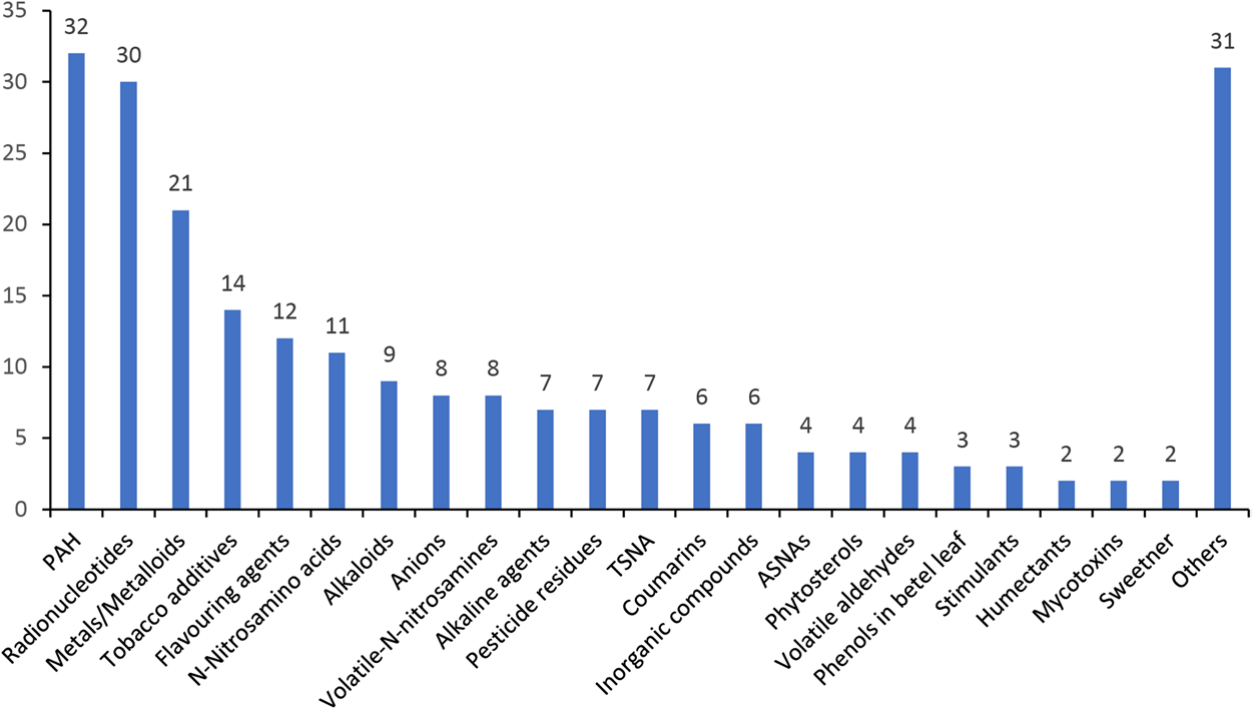
Broad classification of compounds in SLT products as per information retrieved from peer-reviewed literature. *TSNA - Tobacco Specific Nitrosamines, ASNA- Areca-nut Specific Nitrosamines, PAH- Polycyclic Aromatic Hydrocarbons.

Among the 233 identified chemical compounds, major categories included PAH (32) followed by Radionucleotides (30), Metal/Metalloids (21), Additives (14) and others as shown in Fig. 3.

### Database utility

SLTChemDB holds immense utility for various stakeholders viz. researchers and policymakers by providing a one stop information source on chemical constituents of SLT products. SLTChemDB is the first such database that contains raw data useful for tobacco testing laboratories across the globe. Information from the database can also be used to identify the products and brands having minimum concentration of carcinogenic compounds. As an example, among different SLT products amount of NNN varies from 0.0132 µg/g in Rapè tobacco (Brand: Rapé Guarany Cristal) to 3085 µg/g (dry weight) in Toombak (Brand: N/A, Country Sample Source: Sudan). The amount of 4-(methylnitrosamino)-1-(3-pyridyl)-1- butanone varies from as low as 0.004 µg/g (wet weight) in Mawa (Brand: Mawa) to as high as 7870 µg/g (dry weight) in Toombak (Brand: N/A, Country Sample Source: Sudan). The amount of Benzo[a]pyrene varies from <0.0001 µg/g in Chewing tobacco (Brand: Click Eucalyptus) to as high as 0.198 µg/g in Moist Snuff (Brand: Kodiak Premium Wintergreens).

Also, the database highlights research gaps by providing product-wise list of identified chemical compounds. Thus, SLTChemDB will formulate the evidence base and initial data depicting the need for regulation and periodic testing of chemical constituents of SLT products.

## SLTChemDB Web Interface

### Data searching

SLTChemDB has a very simple and user-friendly interface. Extensive search options using various tools have been provided, explained briefly as below:

#### Simple search

This search option allows the users to search SLTChemDB in a very simple way using various keywords. User can search comprehensive information about SLT products and their chemical compounds using the options provided in the Search tab. This function has been depicted in Supplementary Fig. 5.

#### Advanced search

Advance search allows user to build complex queries using logical operators like “AND” and “OR” to search across various fields. The advanced search has been explained in Supplementary Fig. 6.

The available fields for Simple and Advanced search are depicted in Table 2.

**Table 2:** Available fields for Simple and Advanced search in SLTChemDB.

Table	Information Displayed	Description (with example)
Chemical Information	·SLTChemDB ID	Unique ID for every chemical in SLTChemDB database eg 1001
	·Molecular formula	Molecular formula of the chemical eg C6H12O6
	·SMILES structure	Canonical SMILES structure of the chemical eg C1CC(N(C1)N = O)C2 = CN = CC = C2
	·IUPAC Name	IUPAC Name of the chemical eg 3-(1-nitrosopyrrolidin-2-yl)pyridine
	·Compound Name	Full name of the compound eg nitrosonornicotine
	·Molecular weight (g/mol)	Molecular weight of the chemical in g/mol eg 105
	·CTD Database ID	CTD Database ID of the chemical
	·ChEMBL ID	ChEMBL ID of the chemical
	·ChemSpider ID	ChemSpider ID of the chemical
	·PubChem ID	PubChem ID of the chemical
	·Abbreviation	Commonly used abbreviation eg NNN
	·IARC Classification	Group assigned to chemical by IARC eg 1
	·Log P	Log P values of the chemical
	·Group	Chemical group as per literature eg Poly aromatic hydrocarbons
	·Hydrogen bond donors	No. of hydrogen bond that can be donated by the chemical
	·Hydrogen bond acceptor	No of hydrogen bonds that can be accepted by the chemical
	·Polar surface area	Polar surface area of the chemical
	·Number of rotatable bonds	Number of rotatable bonds in the chemical
	·Reference	Reference eg 24011230
	·Product Name	Name of SLT product eg Khaini
SLT Product Composition	·Product Brand	Brand of SLT product eg Skoal
	·Compound Name	Name of chemical eg Nicotine
	·Year of Sample collection	Year eg 1992
	·Country tested	Country name eg India
	·Country sample source	Country name eg India
	·Reference	Reference eg 24011230
pH, Moisture, Free Nicotine	·Product Name	Name of SLT product eg Khaini
	·Product Brand	Brand of SLT product eg Skoal
	·pH	pH eg 7
	·Free nicotine (%)	Numerical value eg 50
	·Moisture (%)	Numerical value eg 51
	·Tobacco content (%)	Numerical value eg 10
	·Country tested	Country name eg India
	·Country sample source	Country name eg India
	·Reference	Reference eg 24011230
Target Information	·Protein name	eg Multidrug resistance protein 1a
	·Organism	eg Rattus norvegicus
	·Reference	eg 24011230
Mode of Intake	·Product Name	eg Khaini
	·Mode of use	eg Chewed
	·Tobacco type	eg Tobacco leaf
ADMET	·AMES toxicity	Mutagenic or Not mutagenic
	·BBB permeability	Readily cross BBB or Poorly distributed in the brain
	·Caco2 permeability	High or Low
	·CNS permeability	Unable to penetrate CNS or Can penetrate CNS
	·CYP2D6 inhibitior	Yes/No
	·Fraction unbound in humans	Numerical values eg 0.21
	·Hepatotoxicity	Likely/Not likely to be associated with disrupted normal function of the liver
	·Intestinal absorption in humans	Good absorption or Poor absorption
	·Max. Tolerated dose in humans	Low/High
	·P-glycoprotein substrate	Likely/Not likely to be a substrate of Pgp
	·Renal OCT2 substrate	Not likely to be an OCT2 substrate
	·Skin Permeability	High/Low
	·Total Clearance	Numerical values eg 0.21
	·VDss in humans	Numerical values eg 0.21
	·Water solubility	Numerical values eg 0.21
Country Information	·Country Name	eg India
	·Income group	eg High
	·WHO FCTC Ratified	Yes/No

#### Structure search

Structure based search allows the user to derive information about chemical compounds by providing its structure either by drawing it or uploading a SMILES structure or mol file. User can select from three search types: Substructure/Exact search, Topological fingerprint-based search and MACCS key based search to generate results. Tanimoto coefficient depicting the structural similarity is displayed against each search result. Structure based search takes place using RD kit^28^. Structures are visualized using JSmol^29^. More information about the results of this search function is explained in Supplementary Fig. 7.

#### Compare results

Using this option, user can compare the composition of different chemicals analyzed between available SLT products, brands and/or countries. This function has been explained in Supplementary Fig. 8 with example.

### Data browsing

The current version of SLTChemDB contains information 233 chemical compounds and 82 SLT products. All information about SLT products and their chemicals is stored in seven tables. The following browse tabs are provided:

- Physicochemical Information: This tab displays the compound name, molecular weight, Log P, hydrogen bond donors, hydrogen bond acceptors, polar surface area and links to other chemical databases of all the chemical compounds.
- Biological information: This tab displays the list of proteins targeted by the of all the chemical compounds.
- Toxicological information: This tab displays AMES toxicity, BBB permeability, Caco2 permeability, CNS permeability, CYP2D6 inhibitor, Fraction unbound in humans, Hepatotoxicity, Intestinal absorption in humans, Max. Tolerated dose in humans, P-glycoprotein substrate, Renal OCT2 substrate, Skin Permeability, Total Clearance, VDss in humans and water solubility of all the chemical compounds.
- Chemical Composition: This tab displays information about the types of SLT products, brands, amount of chemicals, country from which sample was taken, country in which sample was tested along with the reference of the study from which information was taken.
- Product Composition: This tab displays information about the brands, pH, moisture content, tobacco content and free nicotine content of SLT products.
- Country-wise Information: This tab displays information about the availability of SLT products in different countries.

### Help tab

For the first-time user, the help tab can provide comprehensive detail about the methods to search and browse through the website to retrieve the desired information. This tab gives the list of information that can be accessed through the different search functions. Screenshots depicting the help tab are included in Supplementary Figs 1–10.

### Statistics tab

The statistics page contains table depicting the overall statistics of SLTChemDB database. It also contains table with the results of toxicological (ADMET) analysis of chemical compounds. A comprehensive list of all the SLT products and their brands with the number of chemicals identified in each brand.

### Download tab

Option to download all the data in.csv format will be available soon.

### Update of SLTChemDB

The database shall be updated regularly to incorporate newly added research on this topic. Chemico-toxicological information on more SLT products shall also be included, whenever available in an authenticated form. Additionally, the database also provides an option to the user to submit his/her own information using the submission form available at SLTChemDB website. However, such data shall be authenticated by our team before inclusion in the database.

## Discussion

With the widespread use of SLT products, there is an enhanced rate of mortality and morbidity associated with SLT use. Since the health effects of SLT are attributed to its chemical constituents, it is essential to study in detail the chemical profile of various products. This research is hindered by the lack of easily available information on chemical composition of SLT products in a readily usable format. The situation is made more complex by the wide variation in chemical profile across brands of same product and within batches of a brand. Hence, SLTChemDB has been developed as the first comprehensive data repository of chemical, biological and toxicological information about chemical compounds identified across various brands of SLT products.

This database holds promise as an invaluable resource for various stakeholders viz. researchers and policymakers by providing a one-stop information source on chemical profiling of SLT products. For instance, SLTChemDB contains information about 222 Moist Snuff brands. Among them, the number of chemicals identified varies from as high as 44 in Copenhagen to just 1 in other brands like Husky Long Cut Wintergreen, Husky Long Cut Natural etc. With this information, SLTChemDB will highlight the existing gaps in testing of SLT products. Thus, this database is likely to be a valuable resource for the researchers with interest in chemical profiling of SLT products.

Since the levels of various chemicals vary widely between SLT products and also within brands of a particular products^30^, the creation of this database assumes importance being a valuable source for comparison between products and brands of a product. As an example, the amount of N-nitrosonornicotine (NNN) in all the moist snuff samples tested from United States varied from 0.71 µg/g to 64 µg/g across different brands. Within one moist snuff brand from United States, Grizzly, the amount of N-nitrosonornicotine (NNN) ranged between 2.64 µg/g to 11.1 µg/g and while within Skoal it varied from 0.76 µg/g (wet weight) to 42.6 µg/g (wet weight). This information from SLTChemDB shall provide evidence-base to policy makers to form stringent policies on regulation of toxic contents in SLT products. We would like to mention one limitation, that since the information on chemical composition of brands has been retrieved from published literature, a few brands in the database do not carry a name due to lack of this information in the concerned papers^12,31–34^.

SLT research is currently a changing arena with ongoing research on various aspects. SLTChemDB has been developed as an activity of WHO FCTC Global Knowledge Hub on Smokeless Tobacco established in ICMR-NICPR. This database shall be regularly updated to incorporate future information to maintain the comprehensibility of this database. In addition, some future directions may include incorporation of metabolites of chemicals present in SLT products and validation of the toxicological and biological information (estimated *in-silico* till now) through *in-vivo* and *in-vitro* methodologies.

## Methods

### Data collection and compilation

The information about chemical compounds present in SLT products was extracted through extensive search of peer-reviewed literature like papers, reports and monographs from PubMed and Google Scholar using various combinations of keywords (Table 3).

**Table 3:** Keywords used for literature survey.

S.No.	PubMed Search	Google Scholar Keyword
1.	“tobacco, smokeless”[MeSH Terms] AND “chemistry”[MeSH Terms]	Chemical composition of smokeless tobacco [exact phrase]
2.	“tobacco, smokeless”[MeSH Terms] AND (“chemistry”[Subheading] OR “chemistry”[All Fields] OR (“chemical”[All Fields] AND “composition”[All Fields]) OR “chemical composition”[All Fields] OR “chemistry”[MeSH Terms] OR (“chemical”[All Fields] AND “composition”[All Fields]) OR “chemical composition”[All Fields])	Composition of smokeless tobacco products [exact phrase]
3.	“tobacco, smokeless”[MeSH Terms] AND ((“J Mol Catal A Chem”[Journal] OR “chemical”[All Fields]) AND Constituents [All Fields])	SLT Chemicals
4.	“tobacco, smokeless”[MeSH Terms] AND products [All Fields]	Chemicals composition of Smokeless tobacco products [exact phrase]
5.	(“tobacco, smokeless”[MeSH Terms] OR (“tobacco”[All Fields] AND “smokeless”[All Fields]) OR “smokeless tobacco”[All Fields] OR (“smokeless”[All Fields] AND “tobacco”[All Fields])) AND Chemicals [All Fields]	Chemicals composition of SLT products [exact phrase]
6.	(“tobacco, smokeless”[MeSH Terms] OR (“tobacco”[All Fields] AND “smokeless”[All Fields]) OR “smokeless tobacco”[All Fields] OR (“smokeless”[All Fields] AND “tobacco”[All Fields])) AND ((“J Mol Catal A Chem”[Journal] OR “chemical”[All Fields]) AND Profiling [All Fields])	Smokeless tobacco chemicals

*MeSH – Medical subject headings.

**Figure 4:**
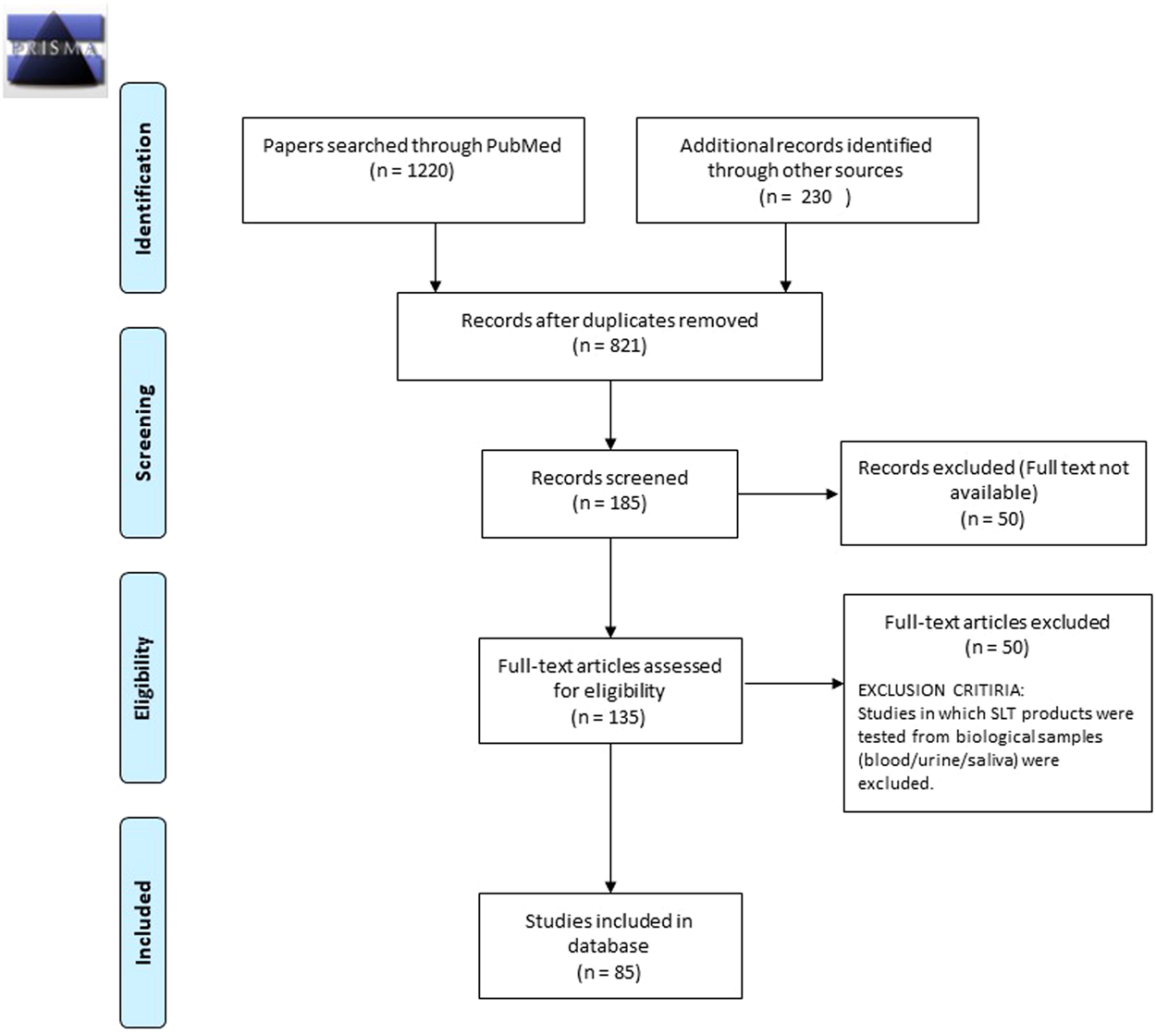
Flow diagram developed using PRISMA depicting data collection process for SLTChemDB. Data collection was finalized on 24 December 2017.

A flow diagram depicting the complete data collection process using PRISMA^35^ is available in Fig. 4.

A total of 821 articles were collected which were filtered on basis of availability of full text and data on testing of SLT products. Information about the classification and composition of chemical compounds along with mode of intake, pH and moisture of SLT products was extracted from 85 published articles.

Data was compiled to obtain information about the physicochemical properties from renowned chemical databases like PubChem^24^ and Chemspider^25^. Further, the information about biological targets was extracted from large scale bioactivity database ChEMBL^26^. Each compound was classified into various carcinogenic groups as per the IARC classification of carcinogens^27–30^. Toxicological information of each chemical compound was calculated using pkCSM server^31^.

### Database framework and web interface

SLTChemDB is developed using efficient and open source technologies like Apache and MYSQL. Front end is developed using HTML, PHP and JavaScript while the back end is supported by PHP. Structure based search takes place using RDKit^28^. 3D structures obtained from existing chemical databases have been converted to 2D using Open Babel^25^. These are further utilized for display and structure-based search. Structures are visualized using JSmol^29^.

### Data organization

#### Primary data

Primary data involves information about classification and composition of chemical compounds along with mode of intake, pH and moisture of SLT products. This information was extracted from peer reviewed published articles.

#### Secondary data

Physicochemical properties of all the identified chemical compounds were extracted from PubChem database. Biological information (protein targets) of each chemical compound was extracted from ChEMBL database. Canonical SMILES structure of chemical compounds taken from PubChem was used for calculation of toxicological properties using pkCSM web server.

## Supplementary information

SLTChemDB: A database of chemical compounds present in Smokeless tobacco products

## Data Availability

The database is freely available at bic.icmr.org.in/sltchem
