# Supplementary material for: SLTChemDB: A database of chemical compounds present in Smokeless tobacco products
Source: Sci Rep. 2019 May 9;9:7142. doi: 10.1038/s41598-019-43559-y (PMC6509116; doi:10.1038/s41598-019-43559-y)
Supplement: Supplementary file 1 — SLTChemDB: A database of chemical compounds present in Smokeless tobacco products [file 41598_2019_43559_MOESM1_ESM.docx]

**SLTChemDB: A database of chemical compounds present in Smokeless tobacco products**

Jasmine Kaur^1$^, Arun Sharma^1$^, Amit Kumar^2^, Deeksha Bhartiya^2^, Dhirendra Narain Sinha^3^, Suchitra Kumari^2^, Ruchika Gupta^4^, Ravi Mehrotra^3,4^, Harpreet Singh^1,^*

Affiliations

^1^Informatics, Systems and Research Management, Indian Council of Medical Research (ICMR), New Delhi-110029, India

^2^Data Management Laboratory, ICMR-National Institute of Cancer Prevention and Research, Noida, Uttar Pradesh-201301, India

^3^WHO FCTC Global Knowledge Hub of Smokeless Tobacco, National Institute of Cancer Prevention and Research, Noida, Uttar Pradesh-201301, India

^4^ICMR-National Institute of Cancer Prevention and Research, Noida, Uttar Pradesh-201301, India

^$^: Equal contribution

*: Corresponding author

Harpreet Singh, Informatics, Systems and Research Management, Indian Council of Medical Research, New Delhi-110029, India

Email: [hsingh@bmi.icmr.org.in](mailto:hsingh@bmi.icmr.org.in)

**
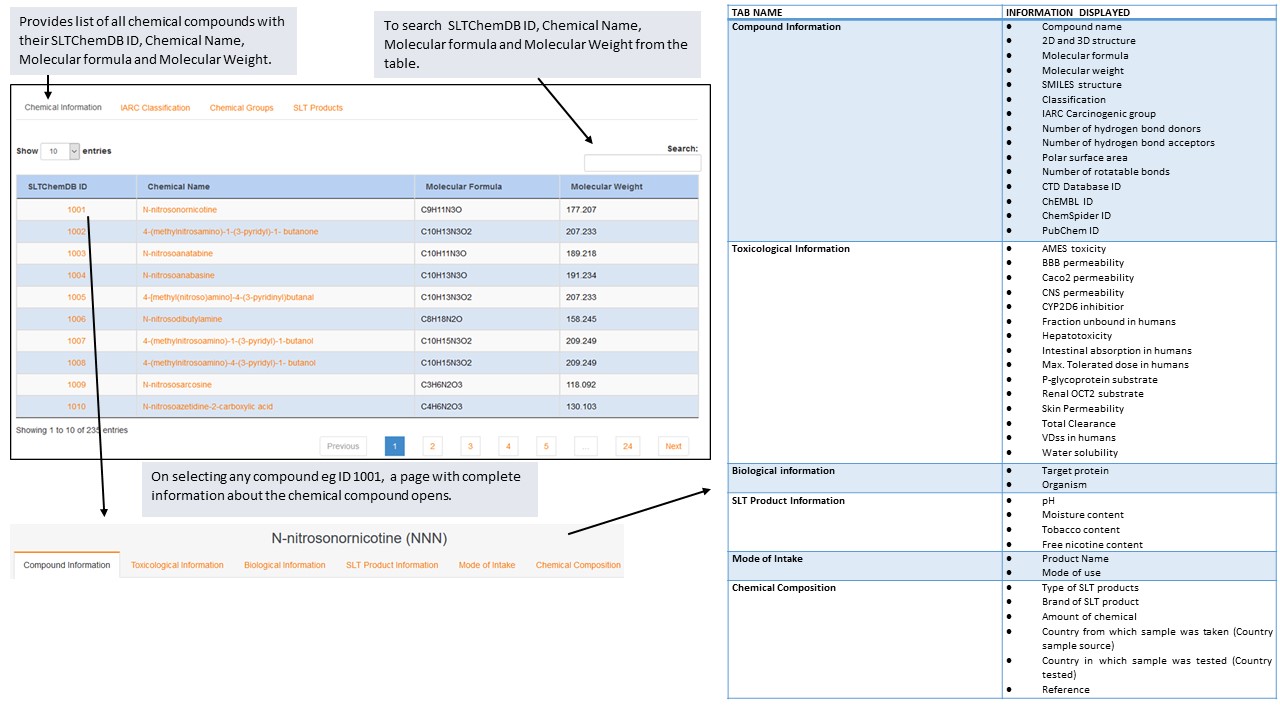
**

**Supplementary Figure 1: Figure depicting “Chemical Information” search option available on the homepage. On selecting any SLTChemDB ID, the comprehensive details obtained are depicted as per Supplementary Figure 10.**

**
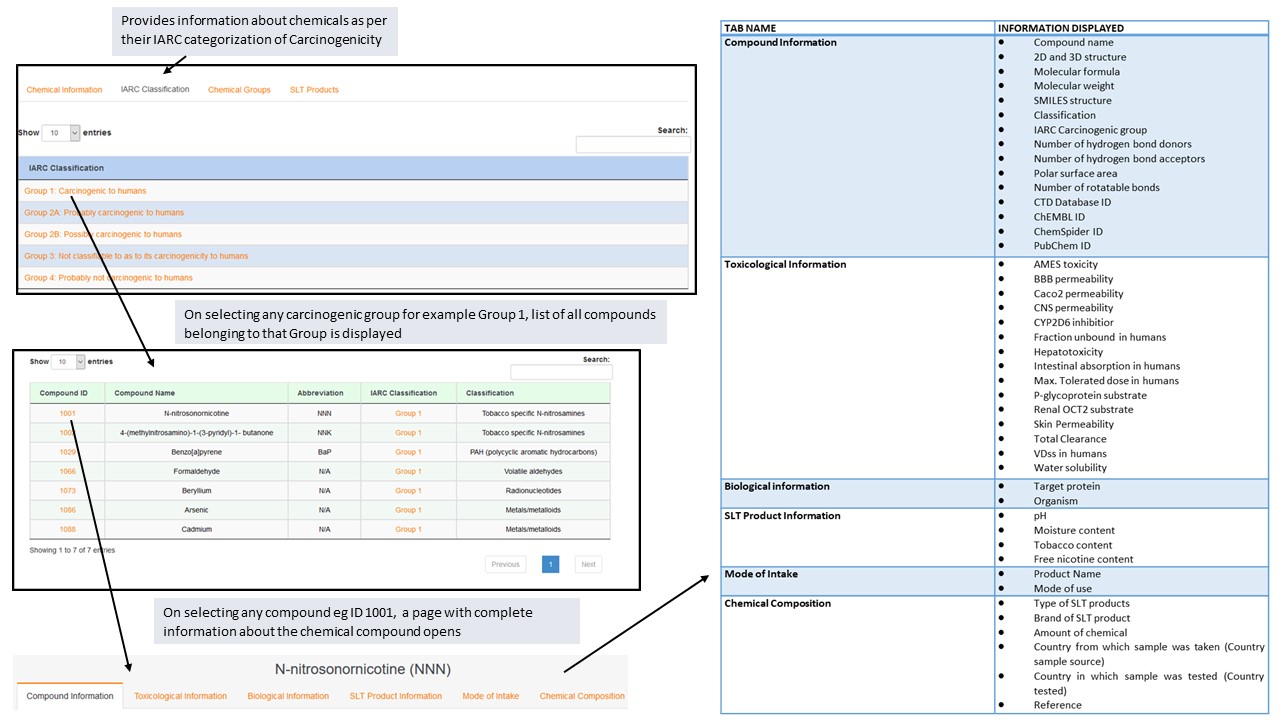
**

**Supplementary Figure 2: Figure depicting “IARC Classification” search option available on the homepage. On selecting any SLTChemDB ID, the comprehensive details obtained are depicted as per Supplementary Figure 10.**

**
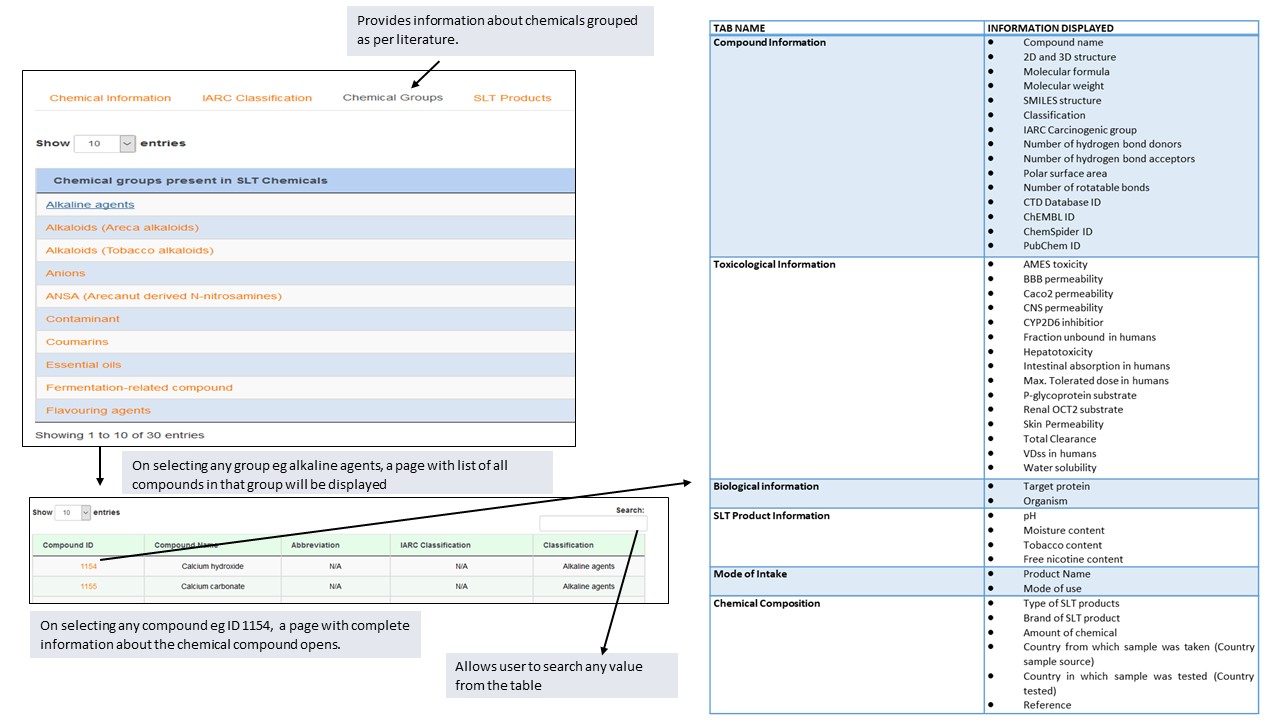
**

**Supplementary Figure 3: Figure depicting “Chemical groups” search option available on the homepage. On selecting any SLTChemDB ID, the comprehensive details obtained are depicted as per Supplementary Figure 10.**

**
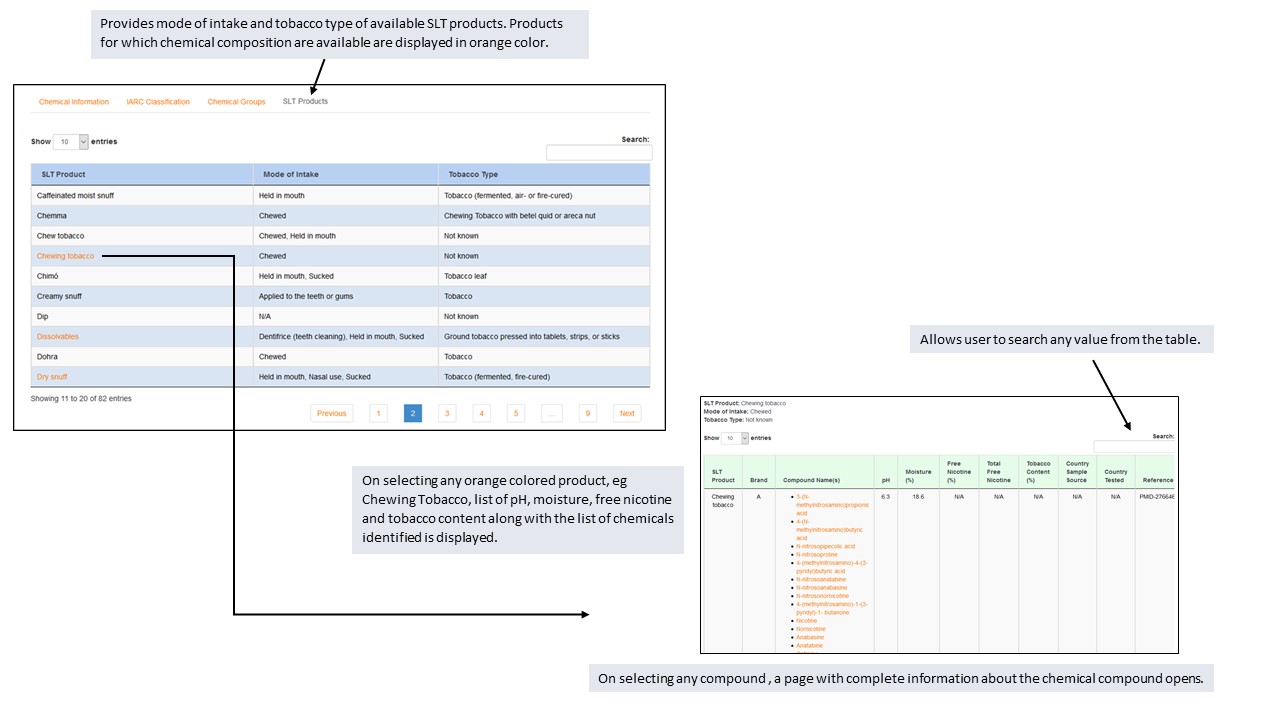
**

**Supplementary Figure 4: Figure depicting “SLT Products” search option available on the homepage. On selecting any SLTChemDB ID, the comprehensive details obtained are depicted as per Supplementary Figure 10.**

**
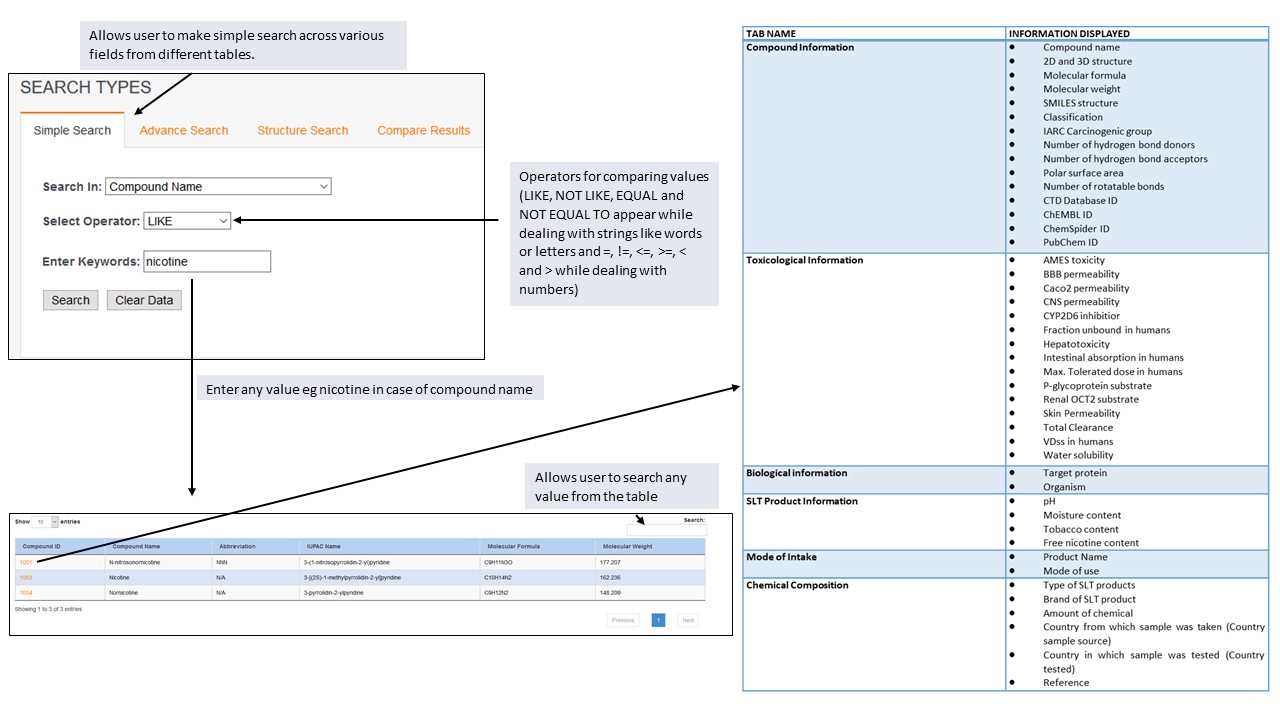
**

**Supplementary Figure 5: Figure depicting Simple search option available in the Search tab. On selecting any SLTChemDB ID, the comprehensive details obtained are depicted as per Supplementary Figure 10.**

**
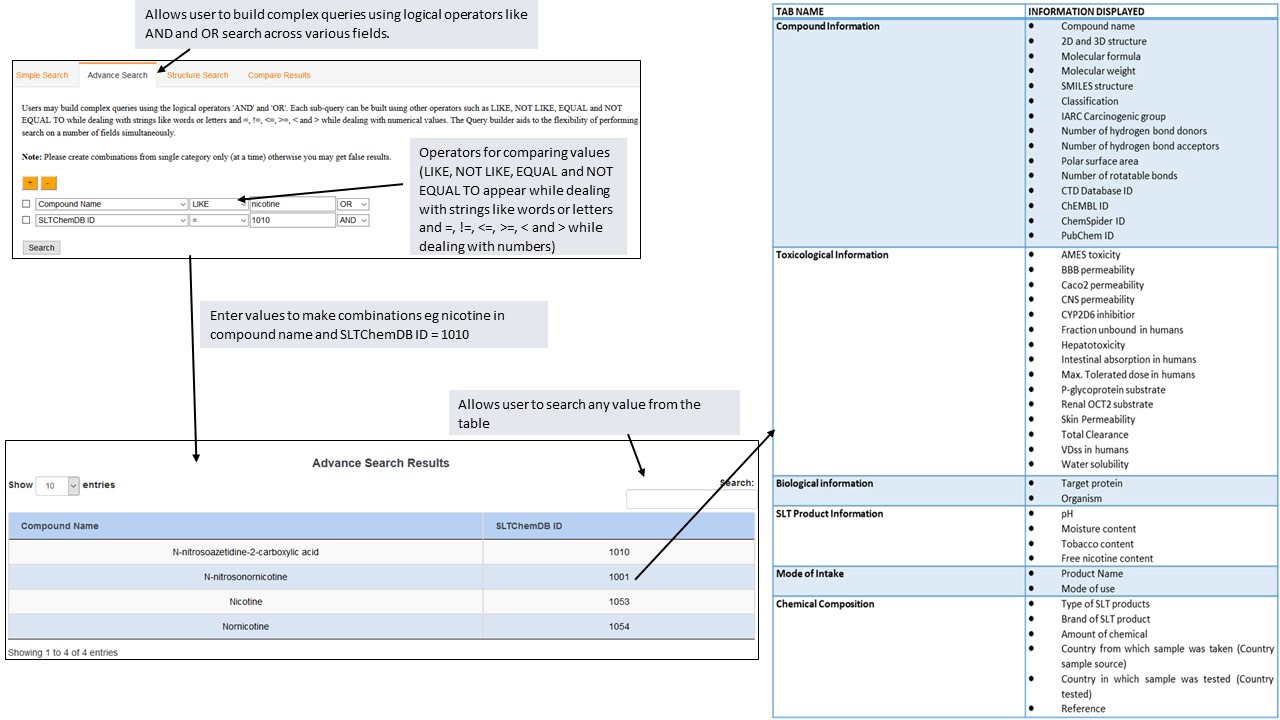
**

**Supplementary Figure 6: Figure depicting Advance search option available in the Search tab. On selecting any SLTChemDB ID, the comprehensive details obtained are depicted as per Supplementary Figure 10.**

**
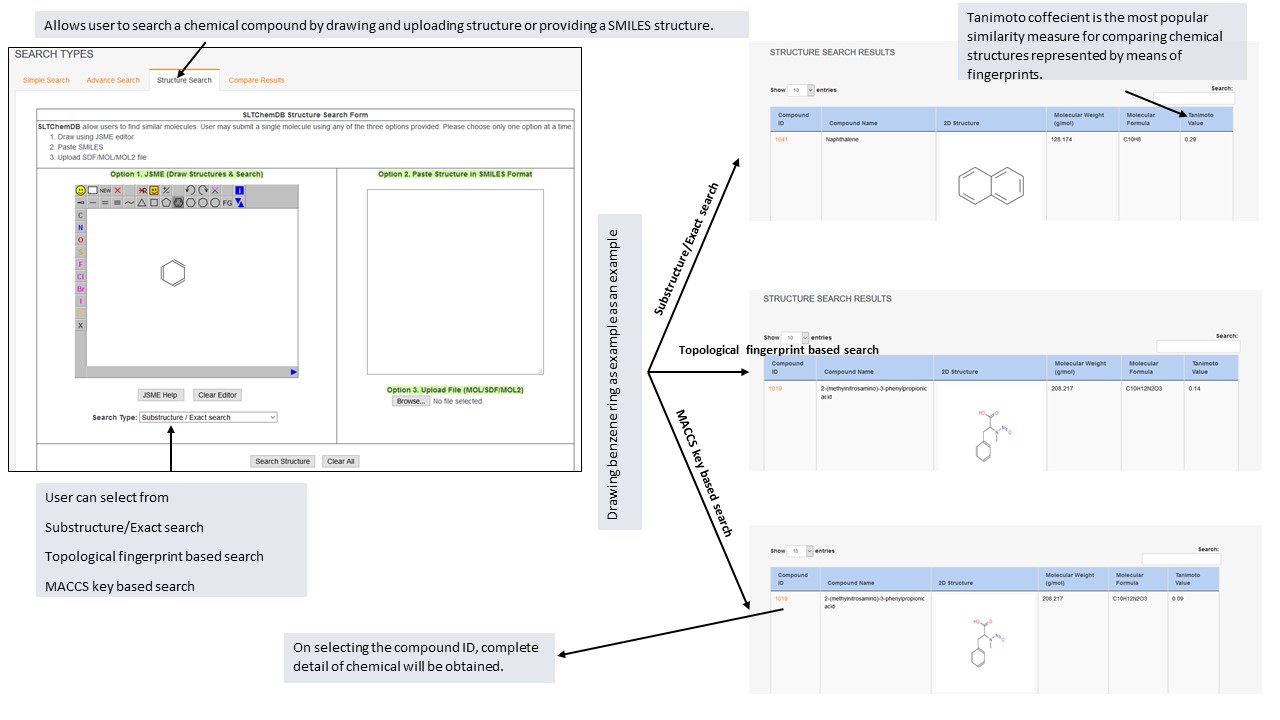
**

**Supplementary Figure 7: Figure depicting Structure search option available in the Search tab. On selecting any SLTChemDB ID, the comprehensive details obtained are depicted as per Supplementary Figure 10.**

**
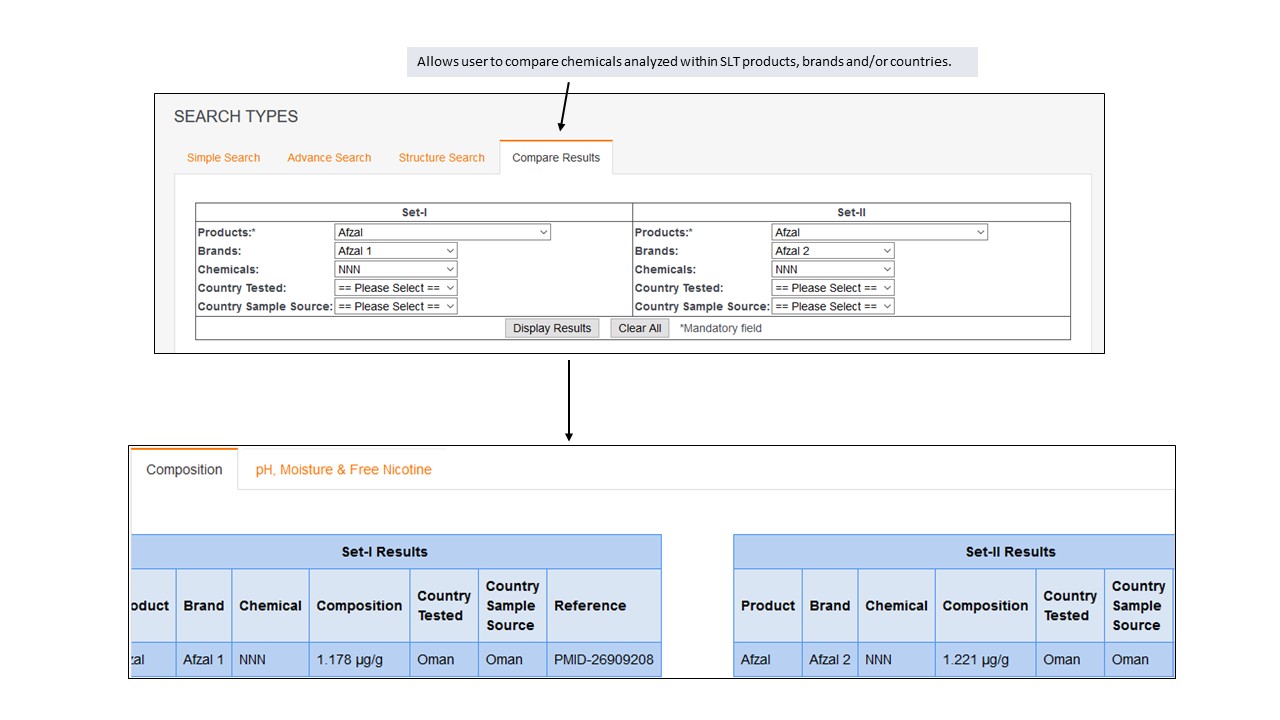
**

**Supplementary Figure 8: Figure depicting Compare Results option available in the Search tab. On selecting any SLTChemDB ID, the comprehensive details obtained are depicted as per Supplementary Figure 10.**

**
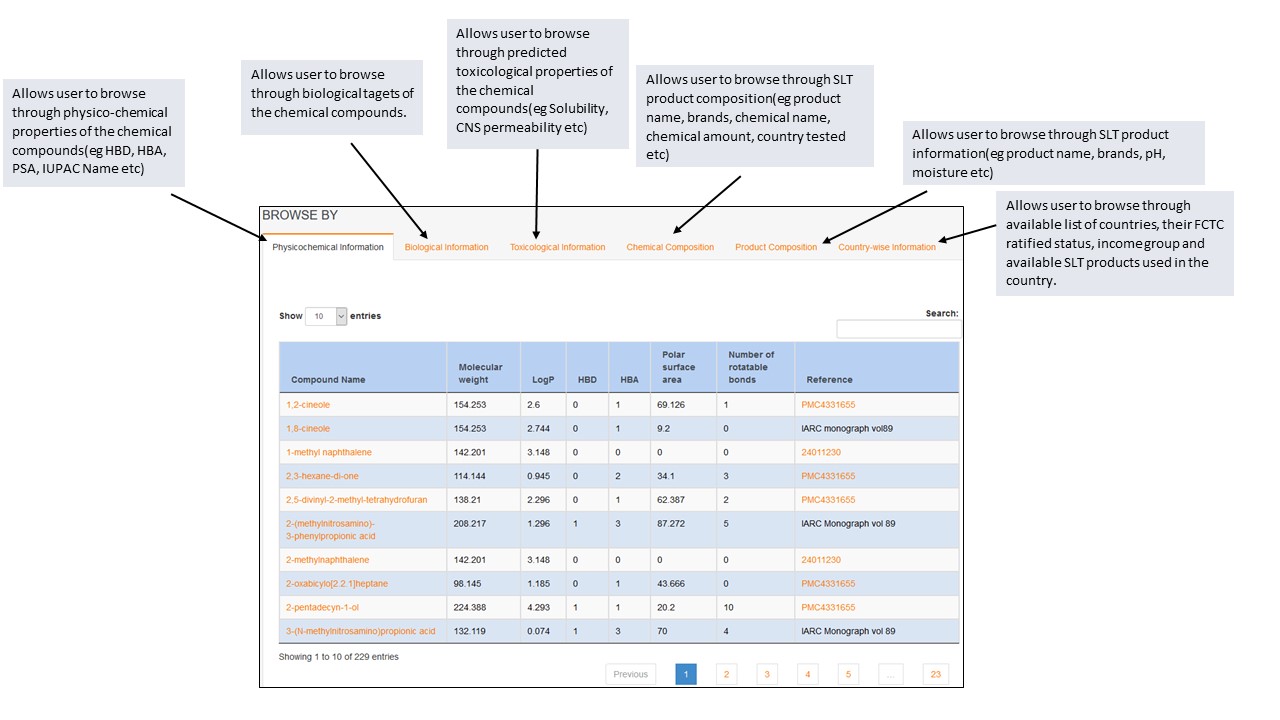
**

**Supplementary Figure 9: Figure depicting all browsing tabs available in the Browse tab. On selecting any compound name, the comprehensive details obtained are depicted as per Supplementary Figure 10.**

**
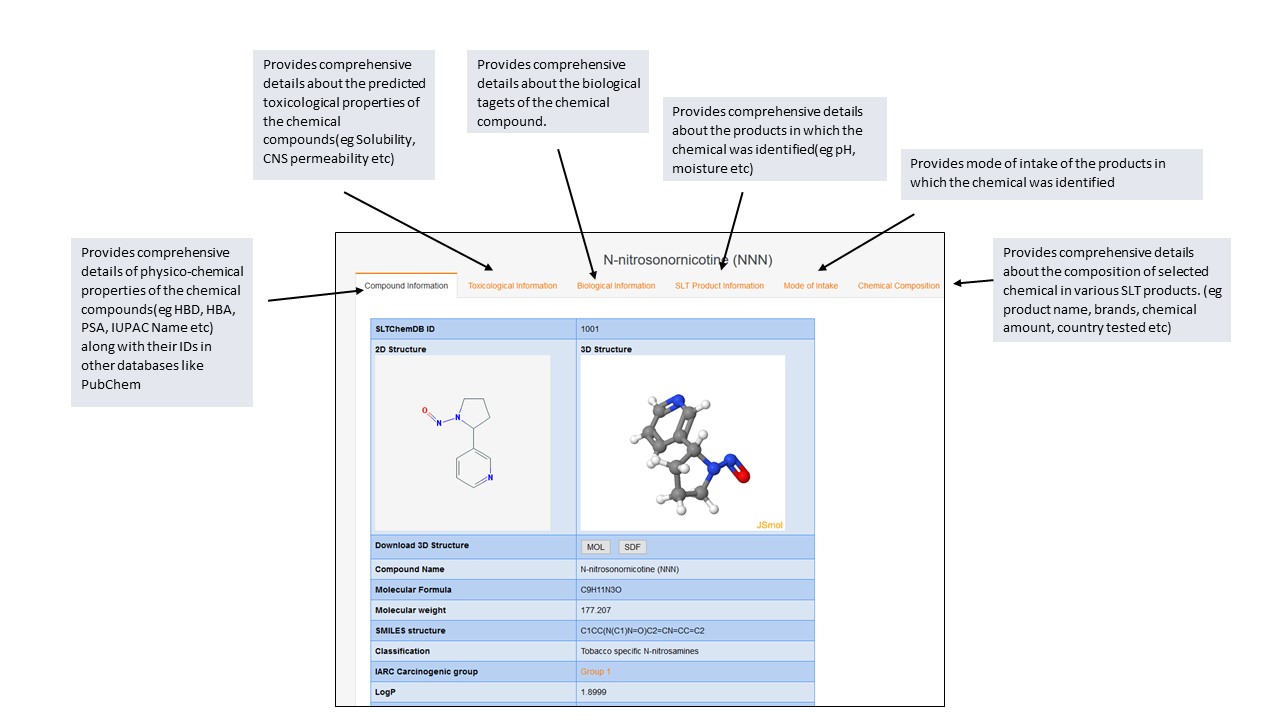
**

**Supplementary Figure 10: Figure depicting comprehensive detail of chemical compound obtained by selecting SLTChemDB ID or Compound name (where ever it is hyperlinked).**

**Supplementary Table 1: Table providing detailed source of 3D structures available on SLTChemDB website.**

| S.No. | Source | Count | SLTChemDB IDs |
| --- | --- | --- | --- |
| 1 | 3D structures are taken from PubChem (.sdf downloaded and converted to .mol for display on the website) | 167 | 1001, 1002, 1003, 1004, 1006, 1007, 1008, 1009, 1010, 1011, 1012, 1013, 1014, 1015, 1016, 1017, 1018, 1020, 1022, 1023, 1024, 1025, 1026, 1027, 1028, 1029, 1030, 1031, 1032, 1033, 1034, 1035, 1036, 1037, 1038, 1039, 1040, 1041, 1042, 1043, 1044, 1045, 1046, 1047, 1049, 1050, 1051, 1052, 1053, 1054, 1055, 1056, 1057, 1058, 1059, 1060, 1061, 1062, 1063, 1064, 1065, 1066, 1067, 1068, 1069, 1075, 1077, 1078, 1079, 1080, 1081, 1082, 1083, 1084, 1085, 1090, 1092, 1093, 1095, 1096, 1110, 1111, 1112, 1113, 1114, 1115, 1117, 1118, 1119, 1120, 1121, 1122, 1123, 1124, 1125, 1126, 1127, 1128, 1129, 1130, 1131, 1132, 1133, 1134, 1135, 1136, 1137, 1138, 1139, 1140, 1141, 1142, 1143, 1144, 1145, 1147, 1148, 1149, 1150, 1152, 1153, 1155, 1156, 1157, 1159, 1160, 1161, 1163, 1164, 1166, 1167, 1168, 1169, 1171, 1172, 1173, 1174, 1175, 1176, 1177, 1179, 1181, 1182, 1183, 1184, 1185, 1187, 1188, 1189, 1190, 1191, 1192, 1193, 1195, 1198, 1199, 1201, 1202, 1203, 1204, 1205, 1211, 1213, 1217, 1233, 1239, 1243 |
| 2 | 3D structures Not Available/ Could not be downloaded from PubChem so canonical SMILES taken from PubChem converted to .mol for display on the website | 56 | 1021, 1070, 1071, 1072, 1073, 1074, 1076, 1086, 1087, 1088, 1089, 1091, 1094, 1097, 1098, 1099, 1100, 1101, 1102, 1103, 1104, 1105, 1106, 1107, 1108, 1109, 1154, 1162, 1196, 1197, 1200, 1206, 1207, 1208, 1209, 1214, 1220, 1221, 1222, 1223, 1224, 1225, 1226, 1227, 1228, 1229, 1230, 1232, 1234, 1235, 1236, 1237, 1238, 1240, 1241, 1242 |
| 3 | 3D structure downloaded from ChemSpider | 1 | 1005 |
| 4 | Structures are self-drawn (canonical SMILES converted to .mol for display on the website) | 5 | 1019, 1178, 1180, 1212, 1219 |
| 5 | Structures not available (2 mixtures and 4 chemical information not available) | 6 | 1210, 1215 (Mixtures)  1231, 1186, 1151, 1048 (Chemical information not available) |
